# What do international ethics guidelines say in terms of the scope of medical research ethics?

**DOI:** 10.1186/s12910-016-0106-4

**Published:** 2016-04-26

**Authors:** Rosemarie D. L. C. Bernabe, Ghislaine J. M. W. van Thiel, Johannes J. M. van Delden

**Affiliations:** Julius Center for Health Sciences and Primary Care, University Medical Center Utrecht, Huispost Str. 6.131, Heidelberglaan 100, 3584 CX Utrecht, The Netherlands

**Keywords:** Ethics guidelines, Research ethics, Comparison of guidelines

## Abstract

**Background:**

In research ethics, the most basic question would always be, “which is an ethical issue, which is not?” Interestingly, depending on which ethics guideline we consult, we may have various answers to this question. Though we already have several international ethics guidelines for biomedical research involving human participants, ironically, we do not have a harmonized document which tells us what these various guidelines say and shows us the areas of consensus (or lack thereof). In this manuscript, we attempted to do just that.

**Methods:**

We extracted the imperatives from five internationally-known ethics guidelines and took note where the imperatives came from. In doing so, we gathered data on how many guidelines support a specific imperative.

**Results:**

We found that there is no consensus on the majority of the imperatives and that in only 8.2 % of the imperatives were there at least moderate consensus (i.e., consensus of at least 3 of the 5 ethics guidelines). Of the 12 clusters (Basic Principles; Research Collaboration; Social Value; Scientific Validity; Participant Selection; Favorable Benefit/Risk Ratio; Independent Review; Informed Consent; Respect for Participants; Publication and Registration; Regulatory Sanctions; and Justified Research on the Vulnerable Population), Informed Consent has the highest level of consensus and Research Collaboration and Regulatory Sanctions have the least.

**Conclusion:**

There was a lack of consensus in the majority of imperatives from the five internationally-known ethics guidelines. This may be partly explained by the differences among the guidelines in terms of their levels of specification as well as conceptual/ideological differences.

**Electronic supplementary material:**

The online version of this article (doi:10.1186/s12910-016-0106-4) contains supplementary material, which is available to authorized users.

## Background

When doing research ethics, the basic question is always, “which is an ethical issue and which is not?” It is interesting that though existing internationally-known ethics guidelines on human research somehow help, we cannot help but share Hussein and Upshur’s observations, “There are many structural similarities to these varied guidelines, but, more importantly, there is considerable variability and lack of harmonization across the globe” [1]. Up to the present, we know of no literature that attempts to look at the imperatives (i.e., the “oughts”) in the major internationally-known ethics guidelines to allow us some glimpse at what these “structural similarities” are and what areas are lacking in harmonization. The most commonly cited document is Emanuel, Wendler, and Grady’s “An ethical framework for biomedical ethics” [2]. Some sources mistakenly perceive this literature as a “coalescence of current standards in a single source” [3]. If we look at Emanuel, Wendler, and Grady’s article, there are clear indications that though the various guidelines were consulted, the aim of the article is to produce a “broader, systematic, and comprehensive framework” precisely because for them, existing guidelines are neither systematic nor comprehensive enough [2]. Hence, the Emanuel, Wendler, and Grady framework is just that, another framework, i.e., a proposed ethical structure or system that takes into consideration the various imperatives of the various guidelines. The eight principles, i.e., the fundamental truths, of their framework, “are conceptually included in most of the previously mentioned guidance, although existing guidelines do not necessarily include all of them” [2]. As such, there is still the need to comprehensively look into what exactly these international ethical guidelines say on what constitutes ethical issues in human research. Doing so would provide us a glimpse of areas that are widely similar, and hence areas where consensus is present among the international guidelines, and areas where dissimilarities are present, i.e., areas where consensus is lacking. Hence, we not only gain a comprehensive picture, we also get a glimpse of the work that needs to be done in reaching a consensus in research ethics.

## Methods

When choosing the research ethics guidelines to include in this manuscript, we consulted the list of ethics guidelines discussed in section 2, “Codes, declarations, and other ethical guidance for research with humans,” of the *Oxford Textbook of Clinical Research Ethics* [4]. Documents that may provide some ethical guidance but are not necessarily ethical guidelines were excluded, such as the International Conference on Harmonisation Guideline for Good Clinical Practice [5]. This meant that we included five landmark internationally-known ethics guidelines on biomedical research namely, the Nuremberg Code [6], the Declaration of Helsinki [7], the CIOMS International Ethical Guidelines for Biomedical Research Involving Human Subjects (including the explanatory notes) [8], the Council of Europe’s Additional Protocol to the Convention on Human Rights and Biomedicine concerning Biomedical Research (including the explanatory notes) [9], and the Belmont Report. [10] From each guideline, we extracted the imperatives. To cluster these imperatives, we used the ethical framework for biomedical research of Emanuel, Wendler, and Grady. Their framework is made up of principles and benchmarks. Benchmarks refer to the imperatives they used as points of reference or measurement of the principles. We used their framework to either check if an imperative is already stated in their benchmarks, to add the imperative under one of their principles, or to add the imperative in a new cluster. It is important to note that since we used Emanuel et al.’s framework as a heuristic tool, we used their principles not as our assumed fundamental truths but as umbrella headings (which we shall call “clusters”) for the imperatives. We took the liberty to add new clusters as necessary and to minimally rephrase a principle to allow for wider coverage. For example, instead of “collaborative partnerships” we used “research collaboration” and instead of “fair participant selection,” we used “participant selection.” Apart from categorizing the imperatives, we also took note where the imperatives came from, and thus we also gathered the data on how many guidelines support a specific imperative, i.e., if there is a strong consensus among the guidelines (SC; 5/5, 4/5), moderate consensus (MC; 3/5), weak consensus (WC; 2/5), no consensus (NC; 1/5), or if it is a non-guideline-based imperative (NGI; 0/5 [in the case of benchmarks in the Emanuel et al. framework that do not correspond to any of the imperatives from any of the guidelines]).

## Results

In total, we extracted 560 imperatives. We included the imperatives for vulnerable population in general, for persons unable to consent, and for emergency research. After double checking for redundancies and merging similar imperatives, the imperatives were reduced to 386. In total, we have 12 clusters: basic principles^1^; research collaboration; social value; scientific validity; fair participant selection; favorable benefit/risk ratio; independent review; informed consent; respect for participants; publication and registration; regulatory sanctions; and justified research on the vulnerable population. Table 1 shows the imperatives within these clusters and their consensus levels. The latter three clusters were added from the original eight principles of Emanuel, Wendler, and Grady. In the following, we describe the clusters and the imperatives. Note that in a few instances, we reclustered some of the Emanuel et al. benchmarks. Additional file 1 gives an overview of the reclustered benchmarks of Emanuel et al.

**Table 1:** Ethical imperatives and their levels of consensus (Strong Consensus [SC] 4 or 5/5; Moderate Consensus [MC] 3/5; Weak Consensus [WC] 2/5; No Consensus [NC] 1/5; Non Guideline-based Imperative [NGI])

I Comparison of the basic principles (preambles)
1. The interests and welfare of the human being participating in research shall prevail over the sole interest of society or science. WC
2. Respect for persons (autonomy of autonomous agents and protection for those who lack autonomy) WC respect for persons (autonomy for autonomous agents and protection for those who lack autonomy)
3. Beneficence WC
3.1. Do no harm WC
3.2. Maximize possible benefits and minimize possible harms WC
4. Justice as fairness in distribution or what is deserved must be upheld WC
5. It is the duty of the physician to promote and safeguard the health, well-being and rights of patients, including those who are involved in medical research. WC
6. Medical progress is based on research that ultimately must include studies involving human subjects. WC
7. The primary purpose of medical research involving human subjects is to understand the causes, development and effects of diseases and improve preventive, diagnostic and therapeutic interventions WC
8. Medical research should be conducted in a manner that minimizes possible harm to the environment. NC
9. Distinction between therapy and research must be upheld NC
II Research collaboration
1. Involvement of community representatives in planning research, conducting research, disseminating results and use of results to improve health NGI
2. The details of health care services provided to subjects/community/country during and after the trial should be agreed by the sponsor, officials of the host country, other interested parties, and when appropriate, the community from which subjects are drawn. NC
3. Research should be respectful of community’s values, circumstances, culture, and social practices. NC
4. Distribution of tangible benefits such as authorship and intellectual property rights must be fair NGI
5. Discussions on responsiveness should include representatives of stakeholders in the host country NC
6. In case of capacity building, the objectives should be determined via a dialogue between external sponsor and host-country NC
7. For equivalency trials, approval should be dependent on the joint negotiations, planning, and justification of the sponsor and health authorities of the host country NC
III Social value
1. Research’s social value must be enhanced NGI
* Responsiveness*
2. Research must be responsive to health needs and priorities of host country WC
3. For minor research studies whose outcome is scientific knowledge, there must be assurance that scientific knowledge developed will be for the benefit of the population NC
4. Research should have a potential value for the prospective beneficiaries NC
5. Individuals/groups should benefit from the conduct and the results of the research NGI
* Reasonable availability*
6. In some instances, drugs should be made available to subjects post-authorization NC
7. Relevant considerations of reasonable availability include the following:
7.1. Length of time intervention will be made available to subjects, community or population NC
7.2. Severity of medical condition NC
7.3. Effect of withdrawing the study drug NC
7.4. Cost to subject or health service NC
7.5. Question of undue inducement NC
* Social value in resource-poor settings*
8. Research in poor countries whose results are used primarily for the benefit of affluent countries may be characterized as exploitative NC
9. Negotiations on studies in poor countries should include the following:
9.1. Health care infrastructure required NC
9.2. Likelihood of authorization for distribution NC
9.3. Decisions regarding payments, royalties, subsidies, technology and intellectual property; distribution costs NC
10. The development of health-care infrastructure to be used in research and beyond in countries with limited resources must happen at the onset NC
11. For externally sponsored collaborative research where the host country lacks the capacity to assess and ensure scientific quality and/or ethical acceptability, sponsors/investigators must ensure that the research contributes effectively to national or local capacity to design and conduct biomedical research NC
12. In countries with limited capacity of ethical/scientific review, capacity building expected from sponsor dependent on magnitude of research NC
13. Capacity building includes the ff:
13.1. Establishing and strengthening independent and competent ethics review processes and monitoring NC
13.2. Strengthening research capacity NC
13.3. Developing technologies appropriate to health-care and biomedical research NC
13.4. Training of research and health-care staff NC
13.5. Educating the community from which subjects will be drawn NC
14. Obligations of sponsor to provide health care services vary with the circumstances of particular studies and the needs of the host country NC
* Fair benefits*
15. Distribution of fair benefits to the community NGI
IV Scientific validity
* Scientific design*
1. Scientific design and statistical methods satisfy generally accepted standards and achieve research objectives before approval and throughout research SC
2. Study justified by previous studies and current knowledge SC
3. Research on humans only when there is absence of alternatives WC
4. Research offers means for information not otherwise attainable NC
5. Assessment of scientific quality should take account of the following:
5.1. Research design NC
5.2. Objectives NC
5.3. Technical feasibility NC
5.4. Statistical methods NC
5.5. Potential for reaching valid conclusions with the smallest possible number of participants NC
6. Less invasive procedures should be used once they become available NC
7. Study results must be interpretable and useful in the context of health problem NGI
8. Research design must be feasible given the context NGI
* Protocol*
9. Design and performance of study must be justified in the protocol WC
10. For multi-center research, any change in the protocol should be made at every collaborating center, or there must be an explicit inter-center comparability procedure NC
* Professionalism*
11. Research conducted only by scientifically qualified persons. Highest degree of skill and care required through all stages MC
12. Physicians who supervise patients must be competent and qualified NC
13. The current state of the art of scientific knowledge and clinical experience determine the professional standards and skills expected of professionals in the research NC
14. Investigators should not enter into agreements that unduly interfere with their access to data, ability to analyze data independently, to prepare manuscripts, or to publish them NC
* Use of comparator*
15. Exception to the rule that study drug must be compared with established effective intervention: when established effective intervention is not available and is not likely to be available in the country. NC
16. It is scientifically and ethically preferable to conduct equivalency trials in countries where established effective intervention (as control) is already available. NC
17. For equivalency trials, in the event that established effective intervention is not available and will not be available in the host country, there must be assurances that investigational intervention will be made reasonably available in host country or community NC
18. Studies that do not compare a study drug with an established effective intervention are allowed given the following:
18.1. Responsiveness to health needs of population NC
18.2. When marketing authorization is secured, drug will be reasonably available to the population NC
18.3. Scientific and ethics committees satisfied that the use of established effective intervention would not yield scientifically reliable results relevant to the study population NC
* Issues with placebo*
19. Placebo may be used given the following:
19.1. No proven intervention exists MC
19.2. Participants on placebo not subject to additional risks or serious or irreversible harm WC
19.3. Participants exposed to at most temporary discomfort or delay in relief of symptoms (acceptable risk) WC
19.4. Extreme care to avoid this option NC
19.5. Use of comparator would not yield scientifically NC reliable results
20. Ethical acceptability of placebo trials increases as time decreases and change to active treatment (escape treatment) possible in case of intolerable symptoms NC
21. Trials with placebo that entail only minor risks (even if noninferiority or equivalency trials are possible) may be ethically acceptable given the following:
21.1. REC must be satisfied that the safety and human rights of subjects are fully protected NC
21.2. Participants are fully informed about alternative treatments NC
* Reexamination*
22. When benefit/risk balance has been disturbed, the trial’s continuation should be reconsidered WC
23. In the event of scientific developments, research should be re-examined by a competent body, REC, or DSMB. NC
24. The purposes of re-examination of research project are the following:
24.1. Research needs to be discontinued or if changes are necessary NC
24.2. Research participants or their representatives need to be informed of developments NC
24.3. Additional consent required NC
V Participant selection
1. Participants are selected to maximize social value and enhance the possibility of benefits to participants NGI
2. Research population is selected to ensure compliance with scientific norms and generate valid and reliable data MC
2.1. Underrepresented groups are given appropriate access to participation WC
2.2. Research should not be offered only to some favored patients (for therapeutic research) or to undesirable persons (for risky research) NC
2.3. The exclusion of groups/communities that might benefit should be ethically justified NC
3. Research population selected to minimize risks to participants NGI
3.1. There should be an order of preference of participants based on ability of members of the class to bear burdens and the appropriateness of placing further burdens on already burdened persons. NC
3.2. For nontherapeutic research with risks, less burdened classes of persons should be called upon first, unless research is directly related to the condition of the more burdened class. NC
4. Groups/communities are selected in such a manner that burdens and benefits are equitably distributed. Deviations from this must be morally justifiable. NC
5. Subjects should be drawn from the qualifying population without prejudice, unless there is a sound scientific reason NC
VI Favorable benefit/risk ratio
* Risks*
1. Risks and burdens minimized SC
2. Various risks to individual subjects and others (physical, psychological, social, legal) delineated and probability and magnitude quantified to the extent possible MC
3. No unnecessary risks WC
4. Risks monitored, assessed, and documented WC
5. Risks that may affect individuals, families, or society (or a part of society) must be taken into consideration NC
6. Method to ascertain risk explicit NC
7. Information on risk must also cover risks related to individual characteristics of the participants such as age, presence of disorders, among others NC
8. Harm to environment must be minimized NC
* Benefits*
9. Various possible benefits to individual subjects delineated and probability and magnitude quantified to the extent possible WC
10. Benefits to participants maximized WC
11. Benefits to other individuals or groups affected must be carefully assessed NC
* Assessment*
12. Risk justified: risk do not outweigh potential benefits (risk to subjects outweighed by anticipated benefit to subject + anticipated benefit to society SC
13. Risk and benefit to subjects are usually considered as carrying special weight NC
14. Interests other than those of the subject may at times justify risks as long as subjects’ rights are protected NC
15. In cases of direct benefit, higher risks may be acceptable provided the risks are proportional to benefits NC
16. When assessing direct benefits, a particular course of action must be judged in the light of the participant’s specific health problem. NC
17. Therapeutic interventions are justified, in light of benefits and risks, that they will at least be as advantageous to subjects as any available alternative. NC
18. Nontherapeutic interventions justified by expected benefits to society NC
19. Risks of nontherapeutic interventions must be reasonable in relation to knowledge to be gained NC
20. Nontherapeutic research must entail no more than acceptable risk and burden to the participant NC
21. In nontherapeutic research, the level of risk and burden to individuals able to consent may be higher compared to those not able to consent NC
22. For nontherapeutic research, when assessing an intervention, it must meet the criteria of relevance and proportionality between the aim pursued and means employed. NC
23. In assessing risks and benefits of a protocol to a population, it is appropriate to consider the harm that could result by foregoing the research. NC
24. Determine if estimates of risks and benefits are reasonable NC
25. Net risk: knowledge to be gained justifies risk NGI
VII Independent review
* Research Ethics Committee (REC) Composition/requirements*
1. Independent and competent MC
2. REC provides reasons for decision WC
3. REC members should declare possible conflict of interest WC
4. REC member with interest on a proposal should not take part in assessment WC
5. REC must be multidisciplinary WC
6. Potential conflict of interest, as well as the perception of it, may be as important as actual conflicts. NC
7. Review process transparent NC
8. REC members who withdraw due to conflict of interest should be allowed to offer comments or respond to questions. NC
9. REC members replaced periodically NC
10. Financial assistance should not be provided directly to the REC to avoid conflict of interest and to safeguard the independence of their review. Instead, funds should be made available to appropriate authorities or to the host research institute. NC
11. Participation of laypersons important; the layperson should not be a healthcare professional nor have experience in carrying out biomedical research NC
12. Thought should also be given to gender and cultural balance in the REC composition. NC
* Rights and responsibilities of RECs*
13. An REC must examine the following:
13.1. Scientific merit: aim study design, safety provisions MC
13.2. risks justified and benefits maximized WC
13.3. if monetary and in-kind recompense constitute undue inducement WC
13.4. IC procedures satisfactory NC
13.5. Subject selection equitable NC
13.6. clear wording of info sheet NC
13.7. when intimidation may be present in securing IC, REC consider whether a neutral 3rd party should seek IC NC
13.8. ethical acceptability of placebo NC
13.9. if procedures are sufficient to verify if subject is capable of consent NC
13.10. In terms of compensation, REC should determine the ff in advance
13.10.1. Injuries for which subjects will receive free treatment and in case of impairment be compensated NC
13.10.2. Injuries for which they will not be compensated NC
14. RECs have the right to report to institutional or governmental authorities any serious or continuing non-compliance. NC
15. Unexpected or unforeseen adverse reactions must be presumed compensable NC
16. When risks are significant, there should be extraordinary insistence by REC on justification NC
*For the investigator/sponsor*
17. Every interventional study should be submitted to an REC for independent examination and approval. MC
18. At the end, final report with summary of findings and conclusions must be submitted to REC. WC
19. Research project should be submitted to the state where research is to take place. NC
20. All information necessary for ethical assessment shall be provided in writing to the REC. NC
21. The following shall be provided to the REC:
21.1. Info about principal researcher NC
21.2. Aim and justification of research NC
21.3. Methods and procedures including statistical and other analytical techniques NC
21.4. Summary of the project NC
21.5. Statement on previous and concurrent submissions of the project NC
21.6. Justification for involving human beings NC
21.7. Inclusion/exclusion criteria NC
21.8. Reasons for the use or absence of control groups NC
21.9. Nature and degree of foreseeable risks NC
21.10. Nature, extent, and duration of interventions NC
21.11. Arrangements to monitor, evaluate, and react to contingencies NC
21.12. Timing and details of info and the means proposed for the provision of info NC
21.13. Documentation for IC/authorization for participation NC
21.14. Arrangements to ensure privacy and confidentiality NC
21.15. Arrangements for information generated during research which may be relevant to the health of the participant NC
21.16. Details of all payments/rewards NC
21.17. Conflict of interest of researchers NC
21.18. Details of potential further uses, including commercial, of results, data, or biological materials NC
21.19. Other ethical issues perceived by researcher NC
21.20. Insurance or indemnity to cover for damages that may arise during research NC
22. REC may request for additional necessary information NC
23. No protocol amendment w/o REC approval NC
24. Unexpected or unforeseen adverse reactions must be reported to the REC for prompt review as they occur. NC
25. REC should be informed of the reasons for premature termination of a project NC
*Externally sponsored studies*
26. Ethical standards outside the country of the sponsor should be no less stringent WC
27. Local or national REC reviewing proposals for an external sponsor should have members who are thoroughly familiar with customs and traditions and sensitive to human rights issues. NC
28. Local RECs are usually not authorized to change doses of drugs, inclusion criteria, or similar modifications. NC
29. Changes recommended/demanded by local RECs should be reported to the sponsor for action to ensure protection of other subjects and validity across sites. NC
30. The REC and the health authorities of the host country should verify if the research is responsive to the health needs and priorities of the host country. NC
31. Ethical reviews should be done in both the country of the sponsor and of the host. NC
32. When sponsor is an international organization, review of protocol must be in accordance with its own independent ethical review of standards and procedures. NC
33. RECs within the sponsoring country/international org should determine the following:
33.1. Scientific methods sound and suitable to the aims of the research NC
33.2. Adequate standards of safety NC
33.3. Sound justification for conducting the study in the host country NC
33.4. Research in compliance with ethical standards of the sponsor country/international organization NC
34. When the host country has a developed capacity for independent ethical review, review in the sponsor country may be limited to ensuring compliance with broadly stated ethical standards. NC
35. When host country does not have a developed capacity for review, full review in the sponsoring country or international organization is necessary NC
36. Multiple reviews should be minimized and reconciled NC
*When deception/incomplete disclosure is involved*
37. When deception is necessary in the research’s methodology, the REC should look into the following:
37.1. Consequences for subjects being deceived NC
37.2. Adequate plan for debriefing subjects (whether and how) results NC
37.3. Justification of sponsor that no other research method would suffice NC
37.4. Justification from sponsor that significant advances could result from the research NC
37.5. That nothing has been withheld that, if divulged, would cause a reasonable person to refuse to participate NC
38. In case of research involving incomplete disclosure, the REC should look at the following:
38.1. Incomplete disclosure is truly necessary in the research NC
38.2. No undisclosed risk that is more than minimal NC
38.3. Adequate plan for debriefing subjects and disseminating results to them NC
39. REC should review and approve all proposals to deceive persons other than the subjects NC
40. In cases when other persons (not the subjects) are to be deceived, REC must determine whether these other persons are similarly entitled to the prompt and honest answering of questions NC
41. When subjects cannot be told that information in the IC has been withheld, explicit REC approval necessary NC
*Studies affecting groups*
42. In studies such as epidemiology or sociology where risks to groups may exist, REC must ensure that interests of all concerned have been given due consideration. NC
VIII Informed consent (IC)
*Culture-appropriate consent*
1. Disclosure forms and procedures are sensitive to culture, language, context SC
2. Recruitment procedures and incentives are consistent with cultural, political, and social practices NGI
3. Mechanisms to symbolize consent are consistent with culture and context NGI
*Securing consent*
4. Consent preferably in writing; if not, must be formally documented and witnessed MC
5. IC a process begun with initial contact and continues throughout the course of the study NC
6. Consent process has three elements, all of which must be given importance: information, comprehension, and voluntariness NC
Information
7. Potential participants must be informed of the following:
7.1. Anticipated benefits SC
7.2. Right to withdraw without reprisal SC
7.3. Aims MC
7.4. Methods/procedures MC
7.5. Sources of funding MC
7.6. Institutional affiliations of researchers MC
7.7. All risks and discomforts that a reasonable person would consider material MC
7.8. Any current alternative interventions MC
7.9. After completion of the study, subjects will at least be given the option to be informed of the findings of the research MC
7.10. Post-study provisions WC
7.11. Rights to refuse to participate WC
7.12. After completion of the study, subjects informed of findings that relate to their individual health status WC
7.13. Subjects have rights to access their data on demand, unless otherwise approved by REC WC
7.14. Provisions to ensure privacy and confidentiality WC
7.15. Possible research uses of the subjects medical records and biological specimens taken in the course of clinical care WC
7.16. Whether commercial products may be developed from biological specimens and whether the participant will receive monetary or other benefits WC
7.17. Extent of investigator’s/sponsor’s responsibility to provide medical services to participants WC
7.18. Explanation of how research differs from routine medical care WC
7.19. Treatment will be provided free of charge for specified types of research-related injuries or complications, nature and duration of such care, who will provide care, and uncertainties if any WC
7.20. Compensation, if any, in case of damage, disability or death WC
7.21. That an REC has approved the protocol WC
7.22. Conflict of interest NC
7.23. Rights and safeguards prescribed by law for the subjects’ protection NC
7.24. Rights and safeguards prescribed by law for those not able to consent to research NC
7.25. That the individual is invited to participate NC
7.26. Reasons for considering the individual suitable for the research NC
7.27. Participation is voluntary NC
7.28. For controlled trials, explanation of the features of the research design (e.g., randomization, double-blinding) NC
7.29. Limits to confidentiality and possible consequences of breaches NC
7.30. Policy on use of results of genetic tests and familial genetic info, and precautions to prevent disclosure of results to others without consent. NC
7.31. Sponsors of the research NC
7.32. Whether it is planned that biological specimens collected in the research will be destroyed at its conclusion, and if not, details about storage and possible future use NC
7.33. Subjects have right to decide about the future use of their biological specimens collected during research, to refuse storage, and to have the material destroyed NC
7.34. Whether investigator is only investigator or both investigator and subject’s physician NC
7.35. Whether or not compensation is guaranteed in the country NC
7.36. Offering the subject to ask questions NC
7.37. Duration of participation (including number and duration of visits NC
7.38. Possibility of early termination of trial or of participation NC
7.39. Compensation for participation NC
7.40. Prospective subjects need to be informed that they do not need to take legal action to secure free medical treatment or compensation for injury NC
8. Information complete, accurate, and not overwhelming. WC
9. Sometimes, it may be advisable to give info sheets NC
10. IC process should not contain words that absolve the investigator from responsibility in case of accidental injury, or would imply that subjects waive their right to seek compensation for impairment, disability, or handicap NC
11. If a person wishes to not receive detailed information on any are, this should be respected so long as he/she has received sufficient info to give IC NC
12. The wish of a participant to not receive info should be recorded NC
13. When incomplete disclosure/deception is involved, subjects must be asked to consent to remain uninformed of the purpose of some procedures until research is completed WC
14. When incomplete disclosure/deception is involved, participants provided with omitted info after the study NC
Comprehension
15. Participants’ comprehension of information must be ensured SC
16. Subjects should have the opportunity to ask questions and receive truthful answers before or during research MC
17. Means must be used to ensure potential participant’s understanding of procedure WC
18. Obligation to ascertain comprehension increases with risk NC
Voluntariness
19. The voluntary consent of the participants must be ensured SC
20. IC must be voluntarily obtained for all biomedical research involving humans WC
21. Participants must be actually free to refuse or to withdraw WC
22. Potential participant must be given enough time to decide WC
23. Prospective subject must not be exposed to undue influence (e.g., asking spouse or community leader to influence decision) WC
24. Payments/services should not lead to undue inducement to participate WC
25. Voluntariness demands that consent is free from coercion and undue influence NC
26. In case of a dependent relationship or duress, IC must be sought by a an independent and qualified individual NC
27. Intimidation in any form invalidates IC NC
28. Especially in nontherapeutic research that present more than minimal risk, sponsor/investigator should avoid undue material inducement NC
Competence
29. When the capacity of a person to give consent is in doubt, arrangements must be in place to verify this capacity NC
30. For studies where some subjects may be rendered incapable of IC, the initial protocol submitted for approval should anticipate that some patients may be incapable of consent and propose a form of proxy consent NC
Renewing consent
31. Promptly renewing of consent necessary when new information may affect the willingness of a participants, when material changes occur in the conditions or the procedures, and also periodically in long-term studies NC
32. Any new info relevant to participation must be conveyed to research participants or their representatives, if applicable, in a timely manner NC
Withdrawal of consent
33. Withdrawal of a patient’s consent must be acted on immediately NC
34. In cases when consent is withdrawn but the abrupt discontinuation of therapy could be hazardous to the patient, the healthcare professional must do the following:
34.1. Explain the risk of discontinuing NC
34.2. Seek consent to continue in the study/treatment NC
Person obtaining consent
35. Person obtaining informed consent must be knowledgeable about the research and is capable of answering questions NC
36. The nature of research, needs of potential participant, national practice, and/or law should determine who should provide information to subjects NC
*Use of data/specimen*
37. IC necessary for research using identifiable human material or data; when consent is impossible or impracticable, permission from REC still necessary. NC
38. Consent forms should include a separate section (or a separate consent form) for subjects who are requested for consent for the use of their biological specimens for research NC
39. Records and specimens of individuals who previously rejected to consent may only be used in public health emergencies NC
40. On secondary use of research records/biological specimens, secondary uses are usually constrained by the conditions specified in the original consent NC
41. It is essential that the original consent process anticipates foreseeable plans for future use NC
42. In terms of consent for secondary use, consent must ask for the following:
42.1. Whether there will be secondary use and whether such use will be limited to the type of study NC
42.2. Conditions under which investigators will be required to contact the research subjects for additional authorization NC
42.3. Investigator’s plan, if any, to destroy or to anonymize the records or specimens NC
42.4. The rights of subjects to request destruction or anonymization NC
*Waiver of IC*
43. Waiver of IC must be considered uncommon and exceptional and must in all cases be approved by REC NC
44. Waiver of signed IC may be granted if:
44.1. No more than minimal risk NC
44.2. Procedures are those for which signed consent forms are not customarily required NC
44.3. Also, when the existence of a signed consent form would be an unjustified threat to the subject’s confidentiality NC
45. Waiver of IC may be granted if:
45.1. No more than minimal risk NC
45.2. IC would make the research impracticable NC
46. Consent for the use of medical records/biological specimens may be waived if:
46.1. Research poses minimal risk NC
46.2. Rights/interests of patients not violated NC
46.3. Privacy and confidentiality or anonymity assured NC
46.4. IC would make the study impracticable NC
47. Refusal or reluctance of individuals to participate is not an evidence of impracticability NC
*Supplementary consent*
48. While supplementary consents or permissions may be obtained, there must be ways to ensure that the individual can still decide independent of spouse or community leader, for example. NC
49. In studies affecting groups (e.g., epidemiology, sociology) where risk to groups may exist, it is advisable supplement IC with community consultation NC
IX Respect for participants
*Participant safety*
1. Health of participant monitored to minimize harms: One of the functions of the Data and Safety Monitoring Board is to protect subjects from previously unknown adverse reactions or unnecessarily prolonged exposure to inferior therapy MC
2. Criteria for changing dose or procedures for stopping the study for the health of the participants must be adequate WC
3. All reasonable means should be taken to ensure safety (that death or disabling injury will not occur) WC
4. To protect participants from injury, disability or death,
4.1. Adequate health-care facilities provided and incorporated in study for the safe conduct of research WC
4.2. Proper preparations made NC
5. In some research, counseling (about acquiring a disease unless they take precautions) for the participants may be necessary NC
6. When prospective or actual subjects are found to have a disease unrelated to the research or cannot be enrolled because they do not meet health criteria, they must be referred to/be advised to obtain medical care NC
7. Research shall not delay nor deprive participants of medically necessary preventative, diagnostic, or therapeutic procedures. Treatment of patient should not be altered in a detrimental manner to facilitate research. NC
8. Participants assigned to control groups shall be assured of proven methods of intervention available in the country or region. NC
9. To minimize risk, when intervention to be tested is designed to prevent or postpone a lethal or disabling outcome, therapy that is known to be superior to the intervention being tested must not be withheld (unless it can be ethically justified) NC
10. An individual may choose to take part in research a number of times or regularly provided that it does not endanger the participant’s health NC
11. All steps taken to assess the state of health of human beings prior to inclusion in research and to ensure that those with increased risk are excluded. NC
12. Where research is undertaken on persons in their reproductive stage, particular consideration must be given to possible adverse impact on current or future pregnancy and the health of the embryo, fetus, or child. NC
*Dissemination of research results to participants/community*
13. Everyone is entitled to know the information collected about one’s health; however, one’s wishes to not be informed shall be respected. NC
14. If research gives rise to information relevant to current or future health or quality of life of research participants, this information shall be offered to them within a framework of health care or counseling NC
15. In some circumstances, the right to know or not to know may be restricted for the patient’s interest or in order to protect the rights of a third party or a specified public interest NC
16. In the course of a study, sponsors should disclose to proper health authorities information that might be of public health concern. NC
17. There must be sufficient care on the manner of disseminating research results to the participants and the community NC
*Privacy/confidentiality*
18. Confidentiality procedures must be effectively implemented WC
19. The confidentiality of the subject’s research data must be protected WC
20. Everyone has a right to respect for one’s private life in relation to information about his/her health NC
*Participant care*
21. Free and appropriate medical treatment for injuries incurred due to research participation MC
22. There must be adequate plans for care for participants after research NC
*Compensation*
23. There must be appropriate compensation for subjects harmed due to research participation; in case of death, compensation goes to dependents MC
24. Subjects must not be required to demonstrate the investigator’s negligence or lack of reasonable skill to claim free medical treatment or compensation. NC
25. A subject who withdraws due to research related reasons (such as unacceptable side-effects) or due to health grounds should be paid in full. NC
26. A subject who withdraws for any other reason other than research or health related should be paid in proportion to the amount of participation NC
27. Subject must not be asked to waive right to compensation NC
28. The payment to a subject who has been removed from a study due to willful noncompliance may be partly or wholly withheld. NC
*Sociological/epidemiological studies*
29. These studies may have the risk of group stigmatization or expose its members to discrimination. The following must be observed:
29.1. Maintain confidentiality during and after the study NC
29.2. Publish resulting data in a manner respectful to all concerned, or in some circumstances not publish at all NC
*Physicians and participants*
30. Clinical professional who supervises the research should always be accessible to the participants and ready to respond to their health concerns WC
31. Physician involved in research must protect the life, health, dignity, integrity, right to self-determination, privacy, and confidentiality of personal info of research subject NC
32. Physicians who combine research and medical care should involve their patients only if
32.1. This involvement may be justified by the potential value of the research NC
32.2. There is good reason to believe that the health of the patients will not be adversely affected NC
X Publication and Registration
*Publication*
1. Conflict of interest, sources of funding, and institutional affiliation must be declared in publications WC
2. Publishers should retract any article that has been subsequently found to contain falsified or fabricated data or has been based on unethical research. NC
3. Complete and accurate research results must be publicly available NC
*Registration in a database*
4. Every research involving human subjects must be registered in a publicly-available database before recruitment NC
XI Regulatory Sanctions
1. Disciplinary sanctions must be used as last resort. Preferred methods include cultivation of atmosphere of mutual trust, education and support to promote the capacity for ethical conduct in research. NC
2. Drug regulatory authorities should consider refusal to accept unethically obtained data submitted in support of a marketing authorization application. However, they should also consider the deprivation of benefit to the intended segment of society. NC
XI Special populations
*Vulnerable population in general*
1. safeguards in place to protect the vulnerable depending on their circumstance (age, gender, economic deprivation, social marginalization, clinical status, etcetera) from being involved in research solely for admin convenience or because they are easy to manipulate. MC
2. Research must be justified by its responsiveness to health needs/priorities of the group. Group stands to benefit from knowledge, practice, or interventions that result. WC
3. Research on vulnerable population justified only when research of comparable effectiveness cannot be carried out on a non-vulnerable group. WC
4. Justification required in inviting vulnerable individuals as research subjects. WC
5. RECs reviewing proposals directed at specific diseases or impairments or involving vulnerable people should invite representatives or advocates. NC
6. Though socioeconomically vulnerable people should not disproportionately carry the burden of research, they shouldn’t also be categorically excluded, especially when research addresses a problem prevalent in the group. NC
7. Overuse of certain groups is unjust. NC
8. Research subjects and other members of vulnerable class must be assured of reasonable access to diagnostic, preventive, or therapeutic products that may become available. NC
*Persons not able to consent (including minors)*
9. Research on a person without the capacity to consent may be undertaken only if all are met:
9.1. Necessary authorization given specifically in writing by the legal representative, taking into account the individual’s previously expressed wishes and objections. SC
9.2. Assent is secured and dissent respected SC
9.3. Research of comparable effectiveness cannot be carried out on individuals capable of consent (i.e., condition necessary characteristic of the research group) MC
9.4. Results of the research must have the potential to produce real and direct benefit NC
9.5. Person undergoing research has been informed of his/her rights and safeguards prescribed by law, unless this person is not in a state to receive info. NC
9.6. An adult not able to consent shall as far as possible take part in the authorization procedure. The opinion of a minor shall be taken into consideration as an increasingly determining factor in proportion to age and degree of maturity. NC
10. Non-therapeutic research may be authorized subject to the following:
10.1. Minimal (low) risk and minimal (low) burden (i.e., medical test standard); any consideration of additional potential benefits of the research shall not be used to justify an increased level of risk or burden WC
10.2. Research aims to contribute through significant improvement in scientific understanding of the individual’s condition, disease or disorder NC
11. When risk is above low on researches on individuals unable to consent, the following must be ensured:
11.1. Research is designed to be responsive to disease or conditions they are particularly susceptible to NC
11.2. Risks are only slightly greater than low-risk NC
11.3. Objective of the research sufficiently important to justify exposure of subjects to increased risk NC
11.4. Interventions are reasonably commensurate to the condition under investigation NC
11.5. REC approval NC
12. For studies with individuals unable to consent, such studies may be approved without special substantive or procedural protective measures if the study is low-risk (routine medical tests standard) NC
13. When subjects initially unable to consent becomes capable of consenting, their consent for continued participation must be secured NC
14. Objection to participation, refusal to give authorization or withdrawal of authorization shall not lead to any form of discrimination against the person concerned, in particular regarding the right to medical care. NC
15. Patients with an acute condition that renders them incapable of giving consent may be eligible for inclusion in a trial in which the majority of the prospective subjects will be capable of consent. NC
*Third party authorization*
16. Third parties are those most likely to understand the incompetent subject’s situation and to act in that person’s best interest NC
17. Person authorized to act on behalf of a subject should be given an opportunity to observe the research NC
18. The guardian asked to give permission should be offered no recompense other than a refund of travel and related expenses NC
19. For individuals physically or mentally unable to consent, and consent from a legal rep cannot be obtained, study may proceed provided that reasons stated in protocol and REC approved. Consent must be secured asap. NC
*Emergency research*
20. REC approval must be secured first prior to initiating such a study. WC
21. In emergency research where prior research is not possible, participants, or their representatives if relevant, should be given all relevant information as soon as they are in the state to receive it, and their consent to continued participation should be obtained as soon as is reasonably possible WC
22. The REC and the investigator must set a day when IC must be secured; beyond this day, participation must be discontinued. NC
23. There must be sufficient effort to locate an individual who can give surrogate consent NC
24. Research of comparable effectiveness cannot be carried out in persons in non-emergency situations NC
25. As much as possible, there must be the attempt to identify a population that is likely to develop the condition to be studied for the possibility of securing the IC of these prospective subjects NC
26. Where appropriate, plans to conduct emergency research without prior consent of subjects should be publicized within the community in which it will be carried out NC
27. If acceptability of research is in question, there must be community consultation NC
28. Research should have substantial community support NC
29. Previously expressed objections shall be respected. NC
30. Nontherapeutic emergency research may be justifiable given the following,
30.1. it aims to contribute through significant improvement in the scientific understanding of the individual’s condition, disease, or disorder NC
30.2. Minimal risk and minimal burden NC
*Pregnant women*
31. Pregnant women should be presumed to be eligible for participation in biomedical research. NC
32. Investigators and ethical review committees should ensure that prospective subjects who are pregnant are adequately informed about the risks and benefits to themselves, their pregnancies, the fetus and their subsequent offspring, and to their fertility. NC
33. Research in this population should be performed only if
33.1. it is relevant to the particular health needs of a pregnant woman or her fetus, or to the health needs of pregnant women in general (broadly understood) NC
33.2. Though the decision about acceptability of risk should be made by the mother as part of the informed consent process, it is desirable in research directed at the health of the fetus to obtain the father’s opinion also, when possible. NC
33.3. Special safeguards should be established to prevent undue inducement to pregnant women to participate in research in which interventions hold out the prospect of direct benefit to the fetus NC
33.4. Where fetal abnormality is not recognized as an indication for abortion, pregnant women should not be recruited for research in which there is a realistic basis for concern that fetal abnormality may occur as a consequence of participation as a subject in research. NC
33.5. Investigators should include in protocols on research on pregnant women a plan for monitoring the outcome of the pregnancy with regard to both the health of the woman and the short-term and long-term health of the child. NC
34. Research on a pregnant woman which does not have the potential to produce results of direct benefit to her health, or to that of her embryo, fetus or child after birth, may only be undertaken if the following additional conditions are met:
34.1. the research has the aim of contributing to the ultimate attainment of results capable of conferring benefit to other women in relation to reproduction or to other embryos, fetuses or children, broadly understood NC
34.2. research of comparable effectiveness cannot be carried out on women who are not pregnant NC
34.3. the research entails only minimal risk and minimal burden NC
35. Where research is undertaken on a breastfeeding woman, particular care shall be taken to avoid any adverse impact on the health of the child NC

Of the 386 imperatives, we see in Table 2 that there is the predominance of the lack of consensus at 72.8 %. SC and MC imperatives comprise 8.2 % of all the imperatives. Among all the clusters, Informed Consent has the most number of SC and MC imperatives put together, comprising 14 of the 32 (43.8 %). The following clusters have no SC or MC imperatives: the basic principles; research collaboration; social value; and publication and registration; and regulatory sanctions.

**Table 2:** Summary of clusters and the level of consensus per cluster

Clusters	SC	MC	WC	NC	NGI
Basic principles (10)			8	2	
Research collaboration (7)				5	2
Social value (25)			1	21	3
Scientific validity (37)	2	2	5	26	2
Fair participant selection (10)		1	1	6	2
Favorable benefit/risk ratio (25)	2	1	4	17	1
Independent review (80)		3	8	69	
Informed consent (98)	5	9	20	62	2
Respect for participants (36)		3	7	26	
Publication and registration (4)			1	3	
Regulatory sanctions (2)				2	
Research on vulnerable population (52)	2	2	6	42	
Total	11 (2.8 %)	21 (5.4 %)	61 (15.8 %)	281(72.8 %)	12 (3.1 %)

### Clusters with at least MC

Table 3 provides a snapshot of the clusters and the corresponding 11 imperatives with SC. In detail, the clusters with at least MC are the following:

1. Scientific validity
  The imperatives may be subclustered as follows: scientific design, protocol, professionalism, use of comparator, issues with placebo, reexamination.
  There is SC that scientific validity entails that the scientific design and the statistical methods of a study must satisfy generally accepted standards and that using these methods and design, the research objectives would likely be met. It also entails that the study must be justified by previous studies and current knowledge (SC); and that the research is conducted only by scientifically qualified persons in terms of both skill and care for the participants (MC). Lastly, in terms of the acceptability of the use of placebo, there is MC that it may be used when no proven intervention exists.
2. Participant selection
  There is MC that the research population must be selected to ensure the study’s compliance with scientific norms and the generation of valid and reliable data.
3. Favorable benefit/risk ratio
  The imperatives may be subclustered as follows: risks, benefits, and assessment.
  That risks must be justified in the sense that the risks to participants are outweighed by the anticipated benefits to them and the anticipated benefits to society is a SC imperative that acts as the overarching principle of this cluster. This may mean several things such as the following: there is SC that risks and burdens must be minimized. Also, to ensure proper assessment, there is MC that the various risks (whether physical, psychological, social or legal) must be delineated and the probability and magnitude of such risks must be quantified as much as possible.
4. Independent review
  By independent review, we refer to the ethical assessment by research ethics committees (RECs; otherwise known as institutional review boards). This cluster may be divided into five subclusters: REC composition and requirements, rights and responsibilities of RECs, responsibilities of the investigator/sponsor, externally sponsored studies, and when deception/incomplete disclosure is involved.
  There is MC that an REC must be independent and competent and that it must examine scientific merit by looking at study design and safety provisions. There is also MC that every interventional study must be submitted by the sponsor/investigator to a REC for evaluation and approval.
5. Informed consent
  There is a wide range of consensus levels in this cluster, the highest being SC. This cluster includes imperatives that relate to an ethical informed consent form and procedure. These imperatives may be subclustered into the following: culture-appropriate consent, securing consent (information, comprehension and voluntariness), renewing consent, withdrawal of consent, use of data/specimen, waiver of IC, and supplementary consent.
  There is SC that the disclosure forms and procedures must be culturally sensitive. This relates to an MC that consent must preferably be secured via writing, i.e., through a signature. Otherwise, consent must at least be formally documented and witnessed. In terms of what the participant must be informed about, there is SC that the participant must be informed about the anticipated benefits of the study and the right to withdraw without reprisal. On the other hand, there is MC that the participant must be informed of the aims; methods/procedures; sources of funding; researchers and their institutional affiliations; all risks and discomforts that a reasonable person would consider material; any current alternative interventions; and that the subjects must at least be given the option to be informed of the findings of the research. In terms of comprehension and voluntariness, there is SC that the investigator must ensure that the participants can in fact comprehend the information that is being provided; and that the participants’ consent is truly voluntary.
6. Respect for participants in general
  The imperatives may be subclustered as follows: participant safety, privacy/confidentiality, participant care, dissemination of research results to participants/community, compensation, physicians and participants, sociological/epidemiological studies.
  There is MC that the health of the participants must be monitored for purposes of minimizing harm. When injuries are incurred due to research participation, according to a MC imperative, participants have the right to receive free and appropriate medical treatment. Related to this is another MC imperative which states that participants who have been harmed due to research participation must be appropriately compensated and in case of death, the compensation must go to the dependents of the participant.
7. Respect for vulnerable populations
  The imperatives may be subdivided into the following subclusters: vulnerable population in general, persons not able to consent (including minors), third party authorization, emergency research, and pregnant women.
  Research on persons who are not able to consent may be ethically justifiable when the following conditions are met: the authorization is provided in writing by the legal representative who ideally takes into account the participant’s prior wishes and objections (SC); assent is secured and dissent respected (SC); and research of comparable effectiveness cannot be carried out on individuals who are capable of giving consent (MC).
  The following must be in place for the involvement of vulnerable individuals in research to be justified: safeguards must be in place to protect the vulnerable from manipulation or from being involved in a study due solely for administrative convenience (MC).

**Table 3:** Clusters and imperatives with strong consensus

1. Scientific Validity: study’s design and methodology must be scientifically valid; study must be justified by antecedent studies and current knowledge
2. Favorable Benefit/Risk Ratio: risks and burdens to the research participants must be minimized; the study’s risks must be justified
3. Informed Consent: informed consent forms and procedures must be sensitive to culture, language, and context; participants have the right to be informed of the anticipated benefits and their right to withdraw without reprisal; the participants’ comprehension of the information must be ensured; that consent is voluntary must be ensured
4. Special Populations: the authorization of the legal representative is necessary in the event that the person concerned is incapacitated to consent; for studies on children capable of assenting, that the children’s assent is secured and dissent respected

### Clusters with NC

After looking at the imperatives with a relatively good level of consensus, it is also worthwhile to look at the other end of the spectrum, i.e., clusters with at most NC. These are Research Collaboration and Regulatory Sanctions. The imperatives of these clusters are as follows:

#### Research collaboration

Research must respect the values, circumstances, culture, and social practices of the community. This can be expressed in various ways such as the following: the involvement of community representatives in the planning and conducting research, as well as in the dissemination of results. Also, the details of the health care services information that is distributed to the participants must be the result of an agreement between the sponsor, the host country officials, and when appropriate, the community involved in the research. Discussions on the responsiveness of a research must include stakeholder representatives from the host country. Capacity building plans in the host country must be the result of a bilateral dialogue between the host country and the external sponsor. Lastly, authorship and intellectual property rights must be fairly distributed.

#### Regulatory sanctions

The preferred methods for regulatory action must be those that cultivate an atmosphere of mutual trust, education, and support. As such, disciplinary sanctions must be used as the last regulatory resort. Also, drug regulatory authorities should consider refusing to accept unethically obtained data, but not without considering the effect of such a refusal.

## Discussion

In the introduction, we stated that we wanted a comprehensive list of imperatives to determine consensus areas and areas where consensus is lacking. In the results section and in Table 1, as a response to the former, we extensively presented the imperatives according to clusters and subclusters. We also showed the level of consensus present in each cluster. We are now able to determine the areas where consensus is high or at least moderate, and hence the areas of similarities between the guidelines, and the areas where consensus is lacking. From Table 2, we see that of the 386 imperatives, only 32 are at least with MC and only 11 have SC. This means that currently, based on at least MC imperatives, consensus is at 8.2 %; and, based on SC imperatives, consensus is at 2.8 %.

We also saw that among the imperatives with at least MC consensus, 14 of the 32 come from the Informed Consent cluster. This is also the cluster with the most SC. This may be an indication that there is most agreement within research ethics on informed consent processes and procedures.

At the other end are the clusters Research Collaboration and Regulatory Sanctions where, at most, NC is present. This means not only that there is least agreement within research ethics on these clusters; it also means that since for each entry, only one guideline provides a contribution, these clusters are also the most neglected.

These findings are important for several reasons: first, having a comprehensive picture of the imperatives from the five international ethics guidelines on researches involving humans provides guidance on the question, “which are ethical issues?” In the field of pharmaceutical clinical trial regulation, for example, drug assessors frequently rely on the general statement in the dossier that the clinical trial has been conducted ethically. Given that there is the current trend of increasingly expecting drug regulators to identify and act upon ethical issues [11], having a comprehensive list such as this would help in identifying and categorizing clinical trial issues as ethical in nature. Second, our findings on the level of consensus provide an idea of the magnitude of work to be done in research ethics in terms of international harmonization and agreement. Third, the findings on the clusters where consensus is most present and those where there is no consensus provide some information of which areas are promising in terms of international harmonization and consensus and which areas need more work either because there is no agreement present or because there is relatively little attention on them.

It is important to note that the limited amount of consensus may have been brought about by the fact that some ethics guidelines are more expounded than others. The CIOMS guideline, for example, provides explanatory notes, while the Helsinki Declaration does not. The level of consensus depends on what is written down; however, it may be reasonable to think that some of the low consensus imperatives may in fact be thought of as specifications of the principles stated in shorter guidelines, i.e., that the amount of consensus may increase if only we see some of the low consensus imperatives as specifications of the more general imperatives. There were also differences in terms of the original intended audience of a guideline. The Belmont Report, for example, was originally meant for scientists, members of IRBs, and Federal employees in the USA [10] while the Declaration of Helsinki is addressed primarily to physicians [7]. The latter is the reason why there is NC, for example, on the imperative, “Physician involved in research must protect the life, health, dignity, integrity, right to self-determination, privacy, and confidentiality of personal info of research subject“.

At the same time, actual disagreements among the guidelines may also partly explain the limited level of consensus. For our purposes, we shall demonstrate using imperatives from Table 1 that weakness in consensus is in fact reflective of the theoretical differences we see in the literature.

First, we see that on the level of principles, there is at most WC. There is WC that “the interests and welfare of the human being participating in research shall prevail over the sole interest of society or science” and there is also WC that principles such as beneficence and justice must be upheld. This means that, two guidelines subscribe to the former principle, and two other guidelines subscribe to the latter principle. After back checking for sources, suffice it to say that there is no overlap between guidelines on these two camps. The WC is in fact reflective of the wider disagreement within biomedical ethics of what justification entails, i.e., must it be a justification from a moral theory of human rights (i.e., the top-down approach) or must it come from a balancing of basic principles (such as the four principles of autonomy, beneficence, justice, and nonmaleficence of Beauchamp and Childress) [12]?

Second, within the cluster Social Value, it is noticeable that both “fair benefits” (i.e., fair distribution of benefits must be determined by the parties involved on a case-by-case basis [13]) and reasonable availability” (i.e., a prior agreement between the trial sponsor and host that the medical product will be made ‘reasonably available’ to the host country or community once the product has been approved [14]) are present. These two concepts refer to the two differing proposals in the literature on how to make research socially valuable to the host country or community [14, 15]. Again, this conceptual or ideological difference explains the lack of consensus, with fair benefits being a NGI, and reasonable available as a NC imperative.

Third, the use of the term “therapeutic research” by imperatives within Table 1 at most garners NC. This is reflective of the discussions in the literature on the acceptability of conflating therapy with research [16–19], i.e., the lack of consensus on any of the imperatives that are hinged on this term is reflective of the disagreement within the literature on the acceptability of therapeutic orientation in research.

The stated differences expounded above in terms of level of detail, intended audience, and actual conceptual/ideological differences among the guidelines are also unavoidable limitations of this manuscript.

## Conclusion

In this manuscript, we have provided a comprehensive view of what the various international ethics guidelines for research involving human participants say. We also identified the level of consensus among the imperatives from these guidelines and we discovered that in the majority of the imperatives, there is no consensus. Of the 12 clusters, Informed Consent has the highest level of consensus and Research Collaboration and Regulatory Sanctions have the least. This somehow provides a preview of which areas need more work in terms of achieving international harmonization. The lack of consensus may at least be partly explained by the differences among the guidelines in terms of their levels of specification as well as conceptual/ideological differences on which the imperatives are hinged on.

## Additional file

Additional file 1: An overview of the reclustered benchmarks of Emanuel et al. (DOCX 13 kb)

## Footnotes

1 This cluster contains the explicitly stated “basic principles” (depending on the guideline, this section may be termed as preambles, general ethical principles, and similar phrasings) of the various ethics guidelines.
