# Supplementary material for: What do international ethics guidelines say in terms of the scope of medical research ethics?
Source: BMC Med Ethics. 2016 Apr 26;17:23. doi: 10.1186/s12910-016-0106-4 (PMC4847236; doi:10.1186/s12910-016-0106-4)
Supplement: Additional file 1: — An overview of the reclustered benchmarks of Emanuel et al. (DOCX 13 kb) [file 12910_2016_106_MOESM1_ESM.docx]

**Annex 1**

**Reclustered benchmarks from the Emanuel et al framework**

| Benchmark | From | To |
| --- | --- | --- |
| Assurance of fair benefits to the community from the conduct and results of the research | Collaborative partnerships (Research collaboration) | Social value |
| Adverse impacts from conducting research minimized | Social value | Favorable benefit/risk ratio |
| For vulnerable participants, safeguards must be in place | Social value | Vulnerable population |
| Right to withdraw without penalty | Respect for participants | Informed consent |
